# Brittle-ductile transition stress of different rock types and its relationship with uniaxial compressive strength and Hoek–Brown material constant (*m*_*i*_)

**DOI:** 10.1038/s41598-023-28513-3

**Published:** 2023-01-21

**Authors:** Seyed Morteza Davarpanah, Mohammad Sharghi, Samad Narimani, Ákos Török, Balázs Vásárhelyi

**Affiliations:** 1 Faculty of Civil Engineering, Department of Engineering Geology and Geotechnics, Budapest University of Technology and Economics, Budapest, Hungary; 2 Department of Mining Engineering, Sahand University of Technology, Tabriz, Iran

**Keywords:** Solid Earth sciences, Engineering, Materials science

## Abstract

Rocks deformed at low confining pressure are brittle, which means that after peak stress, the strength declines to a residual value established by sliding friction. The stress drop is the variation between peak and residual values. But no tension reduction takes place at high confining pressure. A proposed definition of the brittle-ductile transition is the transition pressure at which no loss in strength takes place. However, studies that consider information about the brittle-ductile transition, the criterion's range of applicability, how to determine mi, and how confining pressures affect m_i_'s values are scarce. This paper aims to investigate the link between brittle-ductile transition stress, uniaxial compressive strength and Hoek–Brown material constant (*m*_*i*_) for different kinds of rock. It is essential to accurately determine the brittle-ductile transition stress to derive reliable values for m_i_. To achieve this purpose, a large amount of data from the literature was chosen, regression analysis was carried out, and brittle-ductile transition stress (σ_TR_) was determined based on the combination of Hoek–Brown failure criteria and the recently used brittle-ductile transition stress limit of Mogi. Moreover, new nonlinear correlations were established between uniaxial compressive strength and Hoek–Brown material constant (*m*_*i*_) for different igneous, sedimentary and metamorphic rock types. Regression analyses show that the determination coefficient between σ_TR_ and UCS for gneiss is 0.9, sandstone is 0.8, and shale is 0.74. Similarly, the determination coefficient between σ_TR_ and *m*_i_ for gneiss is 0.88. The correlation between Hoek–Brown material constant (*m*_*i*_) and σ_TR_ was not notable for sedimentary and metamorphic rocks, probably due to sedimentary rocks' stratification and metamorphic ones' foliation.

## Introduction

The brittle-ductile transition and nonlinear deformation behaviors are the prominent characteristics of the rock. Rocks transition in failure mode from localized brittle fracture to non-localized plastic flow. This transition plays a significant role in various geophysical and geological problems. The mechanical behavior of rocks in the brittle-ductile transition region is restricted by strain rate, temperature, effective stress, the microstructure, porosity and mineralogy of the rock and water^1–19^.

Kármán^20,21^ was the first who investigate the influence of the confining pressure on the mechanical behavior of the rock. Kármán investigated the effect of the confining pressure of sandstone and marble. According to the literature, the brittle material becomes ductile due to increasing the confining pressure^5,22–26^. However, some rocks still exhibit brittleness even under high confining pressure at 1000 MPa or above^27^ Wang and Yang^28^ developed a new constitutive model based on Mohr–Coulomb (M–C) by integrating an exponential function of damage variable and confining pressure into the yield criterion to describe the brittle-ductile behavior of the crystalline rocks. Recently, Walton^29^, proposed the ductility index to reflect the brittleness of rock based on m_i_ and (UCS) of intact rock. That is, confining stress at the brittle-ductile transition at ambient temperature under typical laboratory strain rates for a given dry, intact rock material has been proposed as a measure of brittleness and has been shown to depend directly on the (UCS) and m_i_.

There are several technical applications where the mechanical behavior of rocks is quite interesting. Deep tunnels, geological repositories for storing radioactive waste, hydropower projects, and the development and production of reservoir resources that, to a sizable percentage, exist in various rocks are a few examples of such uses. For instance, during the excavation of the underground laboratories of the Jinping II hydropower station, various engineering problems arose, including time-dependent failure, slabbing, and rockburst^30^; the occurrence of these problems influenced the stability of the underground engineering works and was closely related to the brittle-ductile characteristics of the surrounding rocks under the imposed stress regime. Because of this, it is crucial to comprehend how mechanically they behave under appropriate conditions of increased confining pressure^31–33^. Triaxial compression experiments are the most popular technique for examining the mechanical properties of intact rock and gathering information for models calculating the strength and deformability of rock masses. The nonlinear Hoek–Brown failure criterion is a commonly used criterion for jointed rock masses used in several global projects and has been found to generate accurate estimations^34,35^.

Mogi^2,36^ showed that the brittle-ductile transition pressures of silicate rocks are appreciably higher than those of carbonate rocks. This difference between silicate and carbonate rocks suggests that different mechanisms of the brittle-ductile transition exist in different rock types. The transition boundary in carbonate rocks is somewhat different from that in silicate rocks, which is attributed to another transition mechanism. However, Byerlee^4^ discussed this problem based on his measurement of friction of rocks, and he argued that the brittle-ductile transition boundary is independent of rock type. Baud et al.^37^, employed the X-ray tomography imaging technique to investigate the brittle-ductile transition for Indian limestone. Their analyses revealed the development of the shear band through the brittle-ductile transition but no evidence of compaction bands. Wang et al.^38^, defined the brittle-ductile index based on the ratio between the post-peak average softening modulus and the difference between the post-peak average softening modulus and Young modulus. However, research combining data from brittle-ductile transition for determining Hoek material constant (*m*_*i*_), uniaxial compressive strength (σ_c_) and how their values are influenced by confining pressure in the higher region of criterion's range of applicability is lacking^39^.

This research aims to determine the brittle-ductile transition stress based on Hoek–Brown failure criteria and Mogi's equation^2^. In other words, by substituting Mogi's equation^2^ in Hoek–Brown criteria, we have obtained a square equation formula where transition stress can be derived. For this purpose, a large database of different rock types was collected from the literature, and transition stress was calculated for different rock types based on the proposed square equation. Then, new nonlinear correlations between Hoek material constant (*m*_*i*_), uniaxial compressive strength (σ_c_) and transition stress (σ_TR_) for each rock type were established.

## Theoretical background

**Figure 1:**
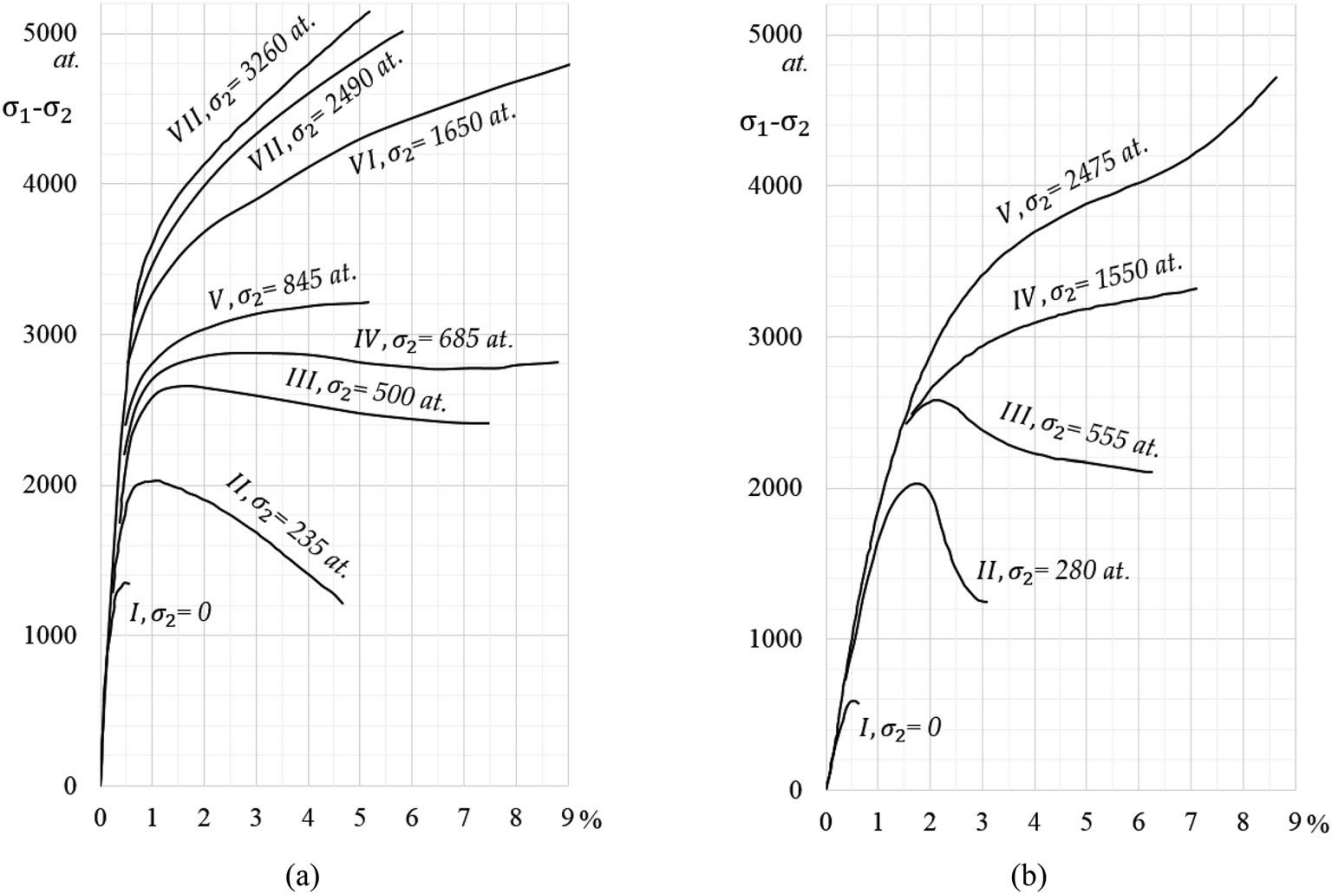
Typical stress–strain curves of (**a**) Carrara marble and (**b**) Mutenberg sandstone in case of different confining pressures (1 atm = 0.101325 MPa)^20,25^.

Some carbonate rocks follow the A-type brittle-ductile transitions, particularly at high temperatures. In contrast, silicate rocks are considered to have B-type stress–strain curves (The typical stress–strain curves of A-type and B-type are schematically shown in Fig. 1a and b, respectively). Thus, the pressure dependence of the strength of rocks near the transition pressure is different between A-type and B-type. Most rocks, however, behave in an intermediate manner between A-type and B-type. An inelastic deformation occurs just before the transition pressure is reached, and after yielding, both fracturing and plastic deformation likely occurs. In addition, it was also suggested that a frictional sliding hypothesis applies to the brittle-ductile transition process of rocks (noted as B-type) in which the permanent deformation in the post-yield region occurs by cataclastic flow or frictional sliding^3^. Also, Kármán^20,21^ published his measured failure limits as functions of the confining pressure. We had to read the data from the figures and recalculate them into MPa—they are collected in Tables 1 and 2, respectively.

**Table 1:** The measured points of failure at the stress space for the marble (recalculated values)^24^.

No. samples	Confining pressure (*σ*_3_ = *σ*_2_) (MPa)	Axial pressure (*σ*_1_) (MPa)
I	0	138
II	24	237
III	51	319
IV	69	361
V	86	411
VI	167	Min. 654
VII	252	Min. 759
VIII	330	Min. 837

**Table 2:** The measured points of failure at the stress space for the sandstone (recalculated values)^24^.

No. samples	Confining pressure (*σ*_3_ = *σ*_2_) (MPa)	Axial pressure (*σ*_1_) (MPa)
I	0	70
II	28	235
III	56	318
IV	157	491
V	251	Min. 717

**Figure 2:**
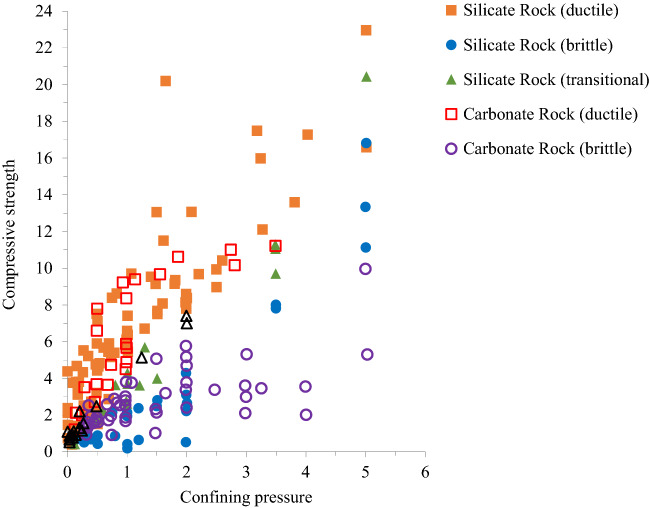
Failure behavior of rocks at various strength and pressure for silicate rocks and carbonate rocks.

**Figure 3:**
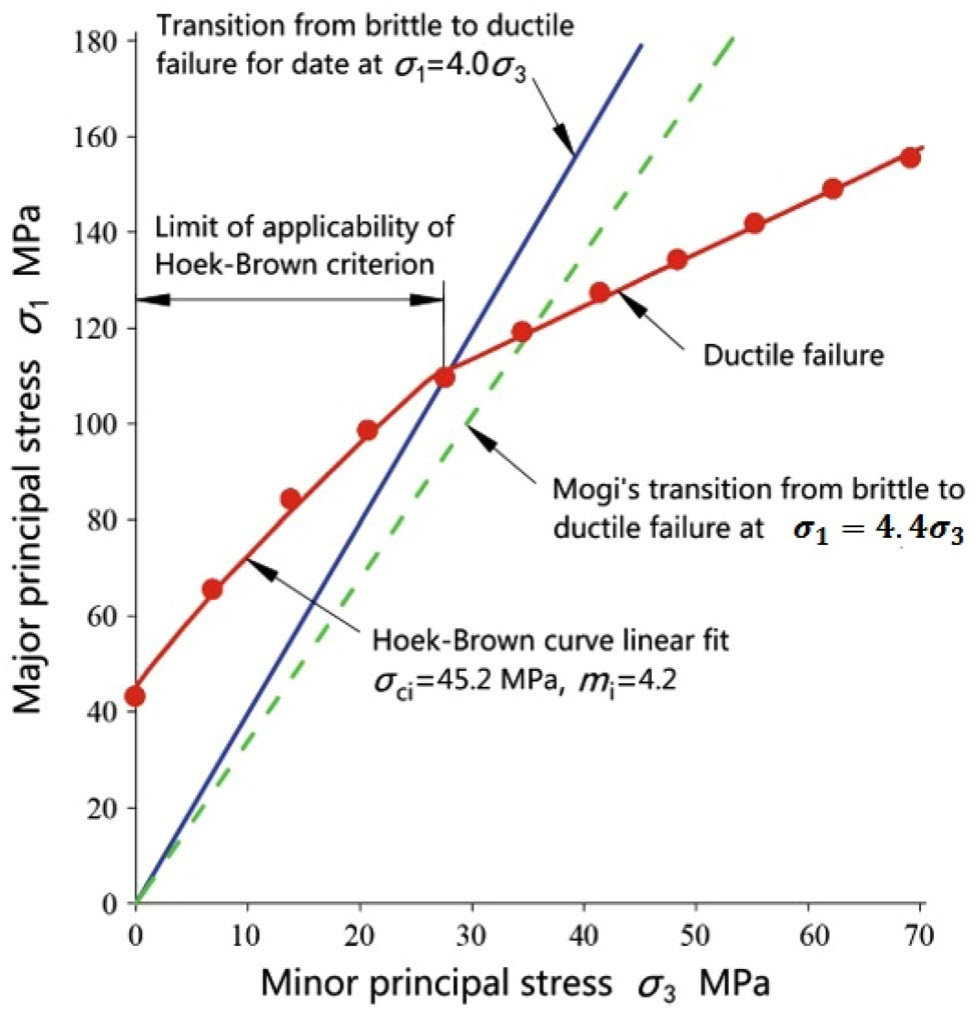
Limit of applicability of the HB criterion^35^.

With the increase of confining pressure, ductility, which is defined as the ability to undergo large permanent deformation without fracture, increases markedly, and a transition from the brittle to the ductile state takes place at some confining pressure^36^. Figure 3 shows the brittle-ductile behavior in the conventional triaxial compression test as a function of the confining pressure and compressive strength of silicate and carbonate rocks given by Mogi^2^. In silicate rocks, the brittle state region and the ductile state region are divided by a straight line passing through the origin (Fig. 2). This boundary line is expressed by (σ_1_ − σ_3_) = 3.4σ_3_.

In this section, to calculate the (σ_TR_), the Mogi ductile–brittle transition stress equation and Hoek–Brown failure criteria are reformulated. The Hoek–Brown (H.B.) failure criterion is widely used in rock mechanics and rock engineering practice. This semi-empirical failure criterion was introduced by Hoek and Brown^40^, and the following form was suggested for intact rock^41^:

1 $${\sigma }_{1}={\sigma }_{3}+{s}_{c}{\left({m}_{i}\frac{{\sigma }_{3}}{{\sigma }_{c}}+1\right)}^{0.5}$$

where ${\sigma }_{1}$ and ${\sigma }_{3}$ are major and minor principal stress at failure, respectively, *m*_*i*_: Hoek–Brown material constant and ${\sigma }_{c}$: the uniaxial compressive strength of intact rock. According to Eq. (1), two independent parameters are necessary, namely the:

- Uniaxial compressive strength of the intact rock (${\sigma }_{c}$),
- Hoek–Brown material constant of the intact rock (*m*_*i*_).

It should be noted that the Hoek–Brown criterion is proposed to deal with shear failure in rocks. Therefore, the Hoek–Brown criterion is only applicable for confining stresses within the range defined by ${\sigma }_{3}$= 0 and the transition from shear to a ductile failure, as shown in Fig. 3. It was indicated that the range of ${\sigma }_{3}$ can significantly influence the calculation of *m*_*i*_^42,43^. Additionally, triaxial test data of Indiana limestone^44^ shows that the applicability of the Hoek–Brown criterion is determined by the transition from shear to ductile failure at approximately ${\upsigma }_{1}=4 {\sigma }_{3}$^35^ (Fig. 3).

**Figure 4:**
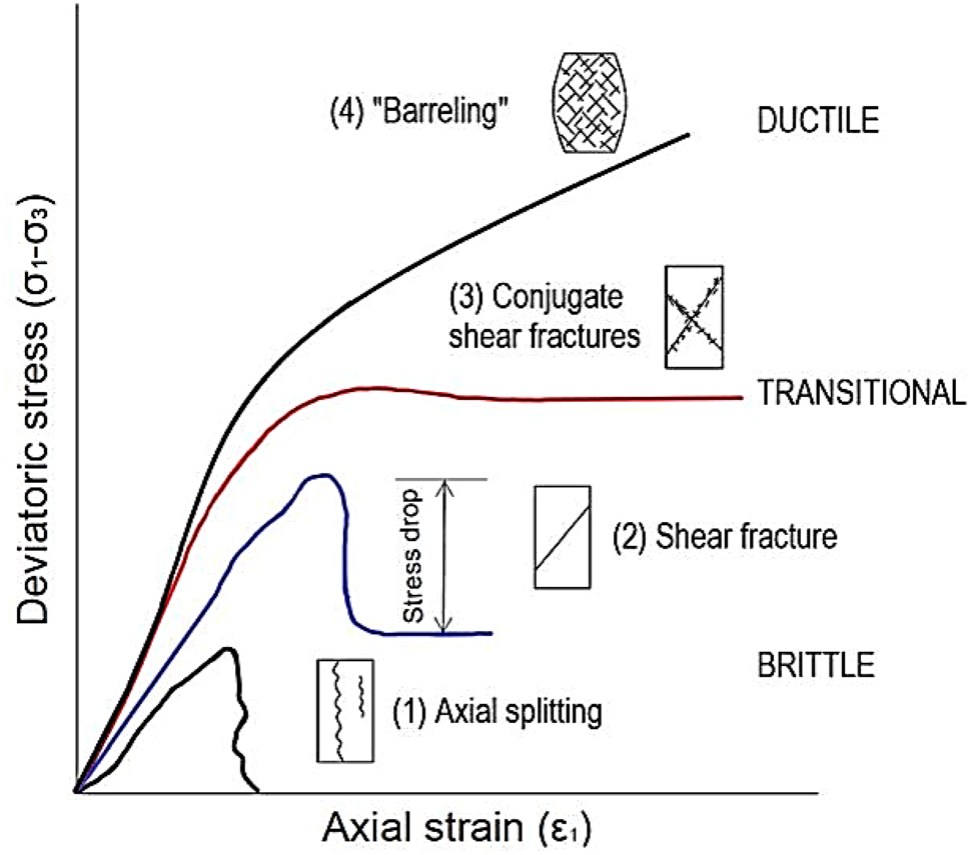
Typical stress–strain curves in brittle, brittle-ductile, and ductile states (modified after^31,47^).

Mogi^2^ found that the average transition is defined as ${\sigma }_{1}=4.4 {\sigma }_{3}$, which is a convenient guide for selecting the maximum confining pressure for triaxial tests of intact rocks. Typical stress–strain curves in the brittle, the transition and the ductile state are very different (see Fig. 4). Brittle rocks break with a slight inelastic strain and a rapid stress reduction after the peak stress, which is referred to as macroscopic failure^5^. The rock exhibits brittle-ductile transition behavior at moderate confining pressures, with a noticeable significant inelastic strain before reaching the peak stress, followed by a slow drop in stress^5,45^. When confining pressures are high, the rock becomes ductile, undergoing a substantial inelastic strain up to peak stress and remaining constant^46^.

**Figure 5:**
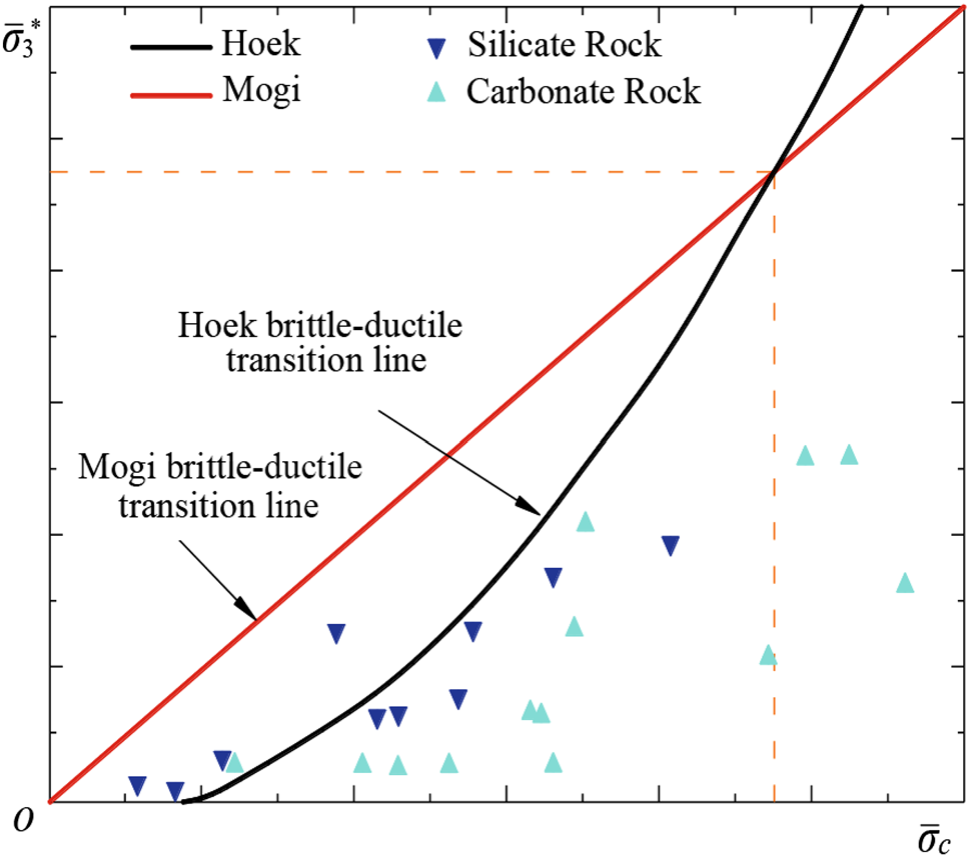
The relationship between the confining pressure at brittleness ductility transition and the value of UCS^48^.

**Figure 6:**
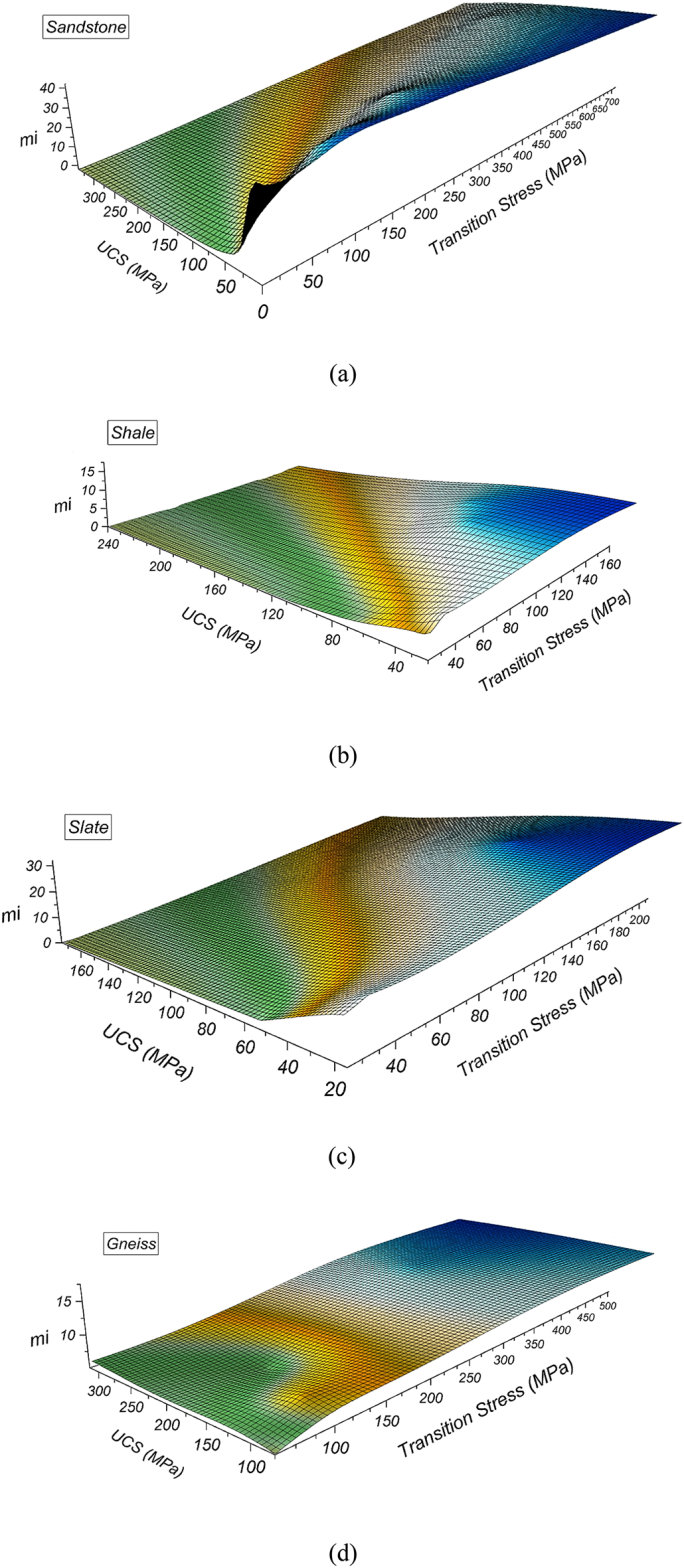
${\sigma }_{TR}$ presented on a color scale as a function of UCS and ${m}_{i}$: (**a**) sandstone; (**b**) shale; (**c**) slate; (**d**) gneiss.

An empirical failure criterion has also been proposed; namely, for most rocks, the confining pressure must always be smaller than the uniaxial compressive strength to keep the brittle behavior of the rock^2^. Figure 5 illustrates the comparison of two criteria [Eqs. (2) and (3)] according to Zuo and Shen^48^. However, most experimental data in Fig. 6 shows that the brittle-ductile transition relationship may be nonlinear. The critical transition condition of brittle-ductile transition for rocks can be expressed by Eq. (2).

2 $${\tilde{\sigma }}_{3}^{*}=\frac{1}{m}\left[\frac{{\tilde{\sigma }}_{c}^{2}}{4b}{\left(\sqrt{1+{m}^{2}}-m\right)}^{2}-b\right]$$

3 $${\tilde{\sigma }}_{3}^{*} \le {\tilde{\sigma }}_{c}$$

In Eq. (2), ${\overline{\upsigma } }_{\mathrm{c}}=\frac{{\upsigma }_{\mathrm{c}}}{{\upsigma }_{\mathrm{t}}}$, $\upmu$ is the friction coefficient, $b$ is the fracture parameter of rocks. Equation (2) indicated that increasing ${\overline{\upsigma } }_{\mathrm{c}}$, the required ${\sigma }_{3}$ to initiate the σ_TR_ increases. Figure 5 illustrates the comparison of two criteria [Eqs. (2) and (3)]^48^.

In this paper, based on the above listed analyses, the transition point from brittle to ductile failure is calculated using σ_TR_ as referred to Mogi's widely used brittle-ductile transition limit for silicate rocks^2^:

4a $${\sigma }_{1}-{\sigma }_{3}=3.4{\sigma }_{3}$$

Thus:

4b $${\sigma }_{1}= 4.4{\sigma }_{3}$$

Substituting Eqs. (4a and 4b) with Eq. (1) we have the following equations:

5 $$4.4 {\sigma }_{3}={\sigma }_{3}+{\sigma }_{c}{\left({m}_{i}\frac{{\sigma }_{3}}{{\sigma }_{c}}+1\right)}^{0.5}$$

${\upsigma }_{3}$ Can be derived from the following equation.

6 $${11.56 \sigma }_{3}^{2}-{m}_{i}{\sigma }_{3}{\sigma }_{c}-{\sigma }_{c}^{2}=0$$

Without taking into account the negative value, the σ_TR_ can be calculated from Eqs. (4a and 4b) using Eq. (6):

7 $${\sigma }_{TR}= {\sigma }_{c}\frac{{m}_{i}+\sqrt{{m}_{i}^{2}+46.24}}{23.12}$$

According to Mogi^2^, for carbonate rocks, the brittle-ductile transition limit (σ_TR_)^2,49^ can be calculated by Eq. (8) for carbonate rocks:

8 $${\sigma }_{TR}= {\sigma }_{c}\frac{{m}_{i}+\sqrt{{m}_{i}^{2}+100}}{50}$$

Incorporating the proposed equations by Davarpanah et al.^39^ for *m*_*i*_ value determination in silicate rocks and carbonate rocks, we have the Eqs. (9) and (10) for estimating σ_TR_, respectively.

9 $${\sigma }_{TR}= {\sigma }_{c}\frac{(\frac{{\sigma }_{c}}{{\sigma }_{t}}-0.17)+\sqrt{{\left(\frac{{\sigma }_{c}}{{\sigma }_{t}}-0.17\right)}^{2}+46.24}}{23.12}$$

10 $${\sigma }_{TR}= {\sigma }_{c}\frac{(\frac{{\sigma }_{c}}{{\sigma }_{t}}-0.17)+\sqrt{{\left(\frac{{\sigma }_{c}}{{\sigma }_{t}}-0.17\right)}^{2}+100}}{50}$$

## Transition stress for different rock types

**Figure 7:**
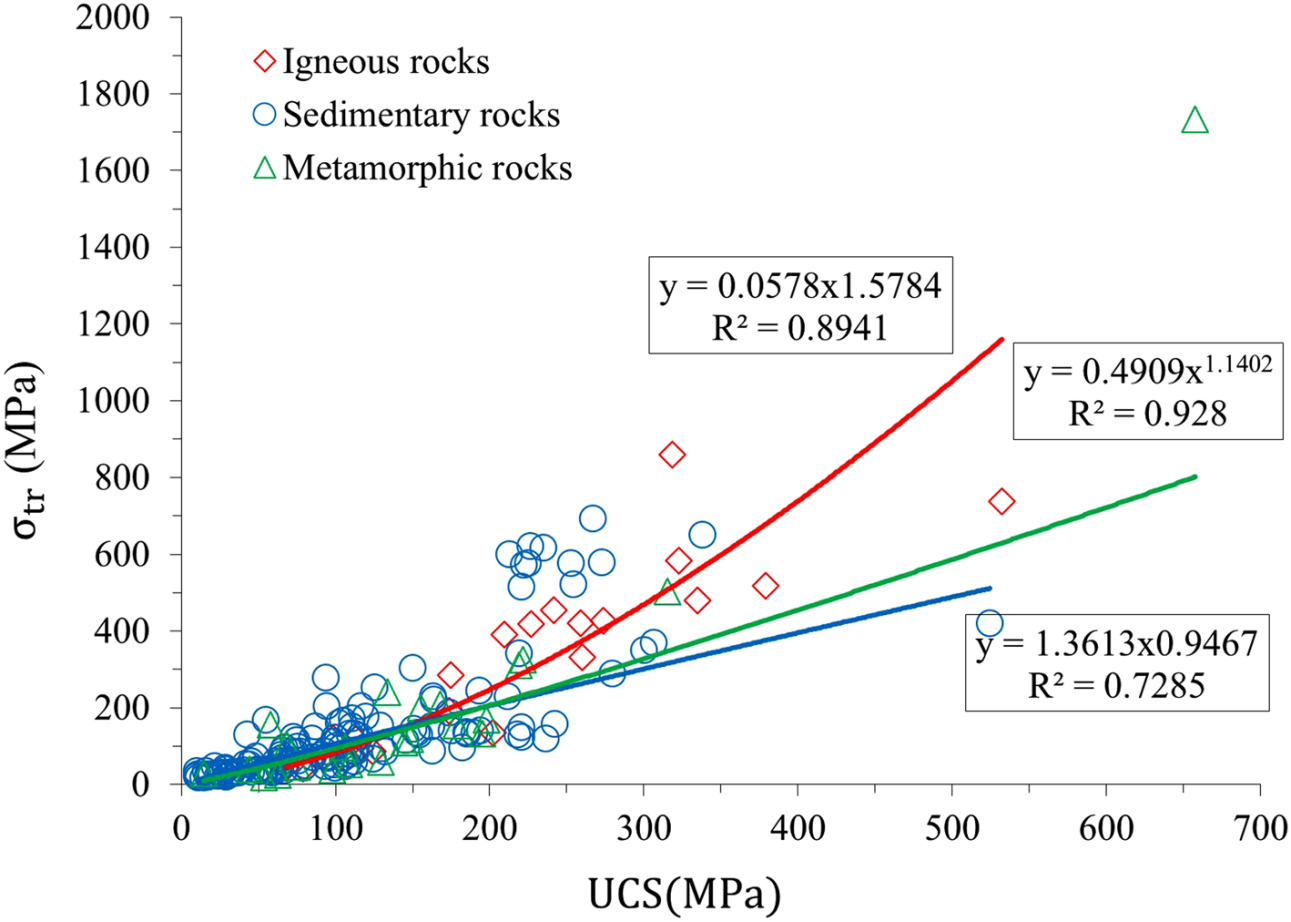
Relationship between ${\sigma }_{TR}$ and UCS.

**Figure 8:**
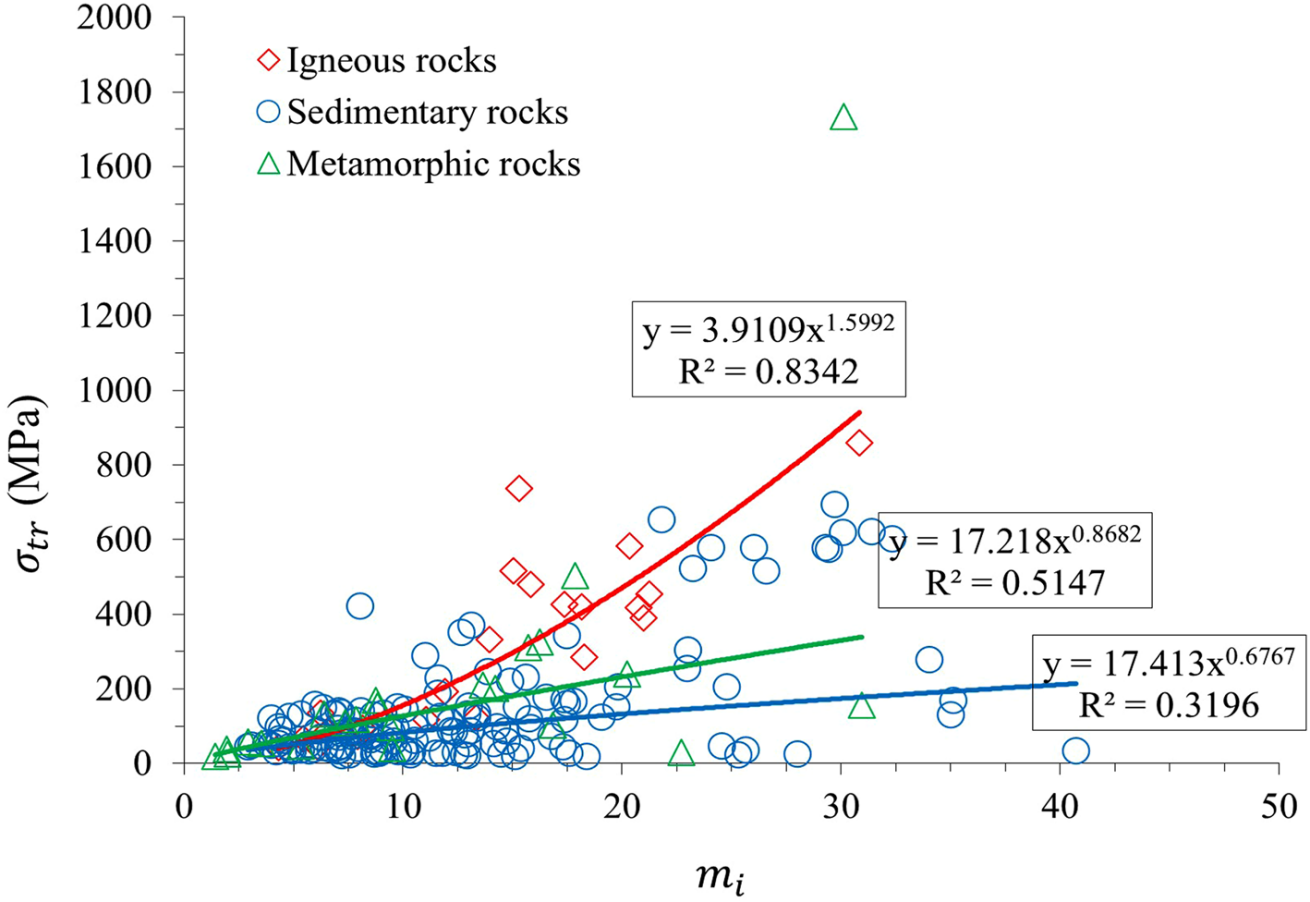
Relationship between ${\sigma }_{TR}$ and *m*_*i*_.

Through collecting the published data by Sheorey^49^, σ_TR_ was calculated for different rock types. The data used in this paper is illustrated in Tables A.1, A.2 and A.3 for igneous, sedimentary, and metamorphic rocks, respectively (see Appendix). The correlations between σ_TR_ and the UCS and *m*_*i*_ are shown in Figs. 6, 7, and 8. As shown in Fig. 8, a high determination correlation was observed for sandstone, shale, and gneiss. (*R*^*2*^ > 0.7); however, the correlation was weak for slate (*R*^2^ < 0.5). Figure 6 shows that by increasing the values of m_i_ and UCS, the values of ${\sigma }_{TR}$ increases; however, the amount of growth depends on the type of rock. For example, according to Fig. 7, for igneous rocks, as UCS increases, the values of ${\sigma }_{TR}$ increases with good data consistency and a high determination coefficient (R^2^ = 0.89). Similarly, for sedimentary and metamorphic rocks, we can see good correlations; however, data consistency is not as significant as for igneous rocks. Figure 8 shows the comparison of the relationship between σ_TR_ and published *m*_*i*_ values. Specifically describing igneous rocks, we can see a strong correlation with a high determination coefficient (R^2^ = 0.83), and good data consistency is notable. Since the constant of mi is an indicator of the brittleness of rock (50), the results show that the influence of m_i_ on ${\sigma }_{TR}$ is more than UCS. All the empirical equations which derived from calculation and correlations are summarized in Table 3.

**Table 3:** Empirical equations derived in this study.

Equation	Rock type	Coefficient of determination (%)
${\upsigma }_{\mathrm{TR}}=0.058{\mathrm{UCS}}^{1.578}$	Igneous rocks	89
${\upsigma }_{\mathrm{TR}}=1.361{\mathrm{UCS}}^{0.947}$	Sedimentary rocks	72
${\upsigma }_{\mathrm{TR}}=0.491{\mathrm{UCS}}^{1.140}$	Metamorphic rocks	68
${\upsigma }_{\mathrm{TR}}=3.911{\mathrm{mi}}^{1.5992}$	Igneous rocks	83
${\upsigma }_{\mathrm{TR}}=17.218{\mathrm{mi}}^{0.868}$	Sedimentary rocks	51
${\upsigma }_{\mathrm{TR}}=17.413{\mathrm{mi}}^{0.677}$	Metamorphic rocks	18

## Discussion

According to our linear and nonlinear regression analyses for different rock types, Fig. 8 shows that σ_TR_ calculated by this research has a high correlation with UCS in most types of rocks, and it can be used to estimate the transition stress of rocks based on their UCS. Figures 7 and 8 indicate that the best correlation was observed for igneous rocks and the reason is more probably related to the texture and the origin of the igneous rocks. A transition to ductile flow is predicted to occur when the strength as a function of pressure (or mean stress) has a slope that deviates from the relatively steep slope in the brittle faulting regime^2^. Implementing such criteria can be ambiguous since the "strength" in the ductile regime evolves with strain hardening and is not well defined. Accordingly, one has to arbitrarily assign it to be the stress attained at a fixed percentage of strain.

**Figure 9:**
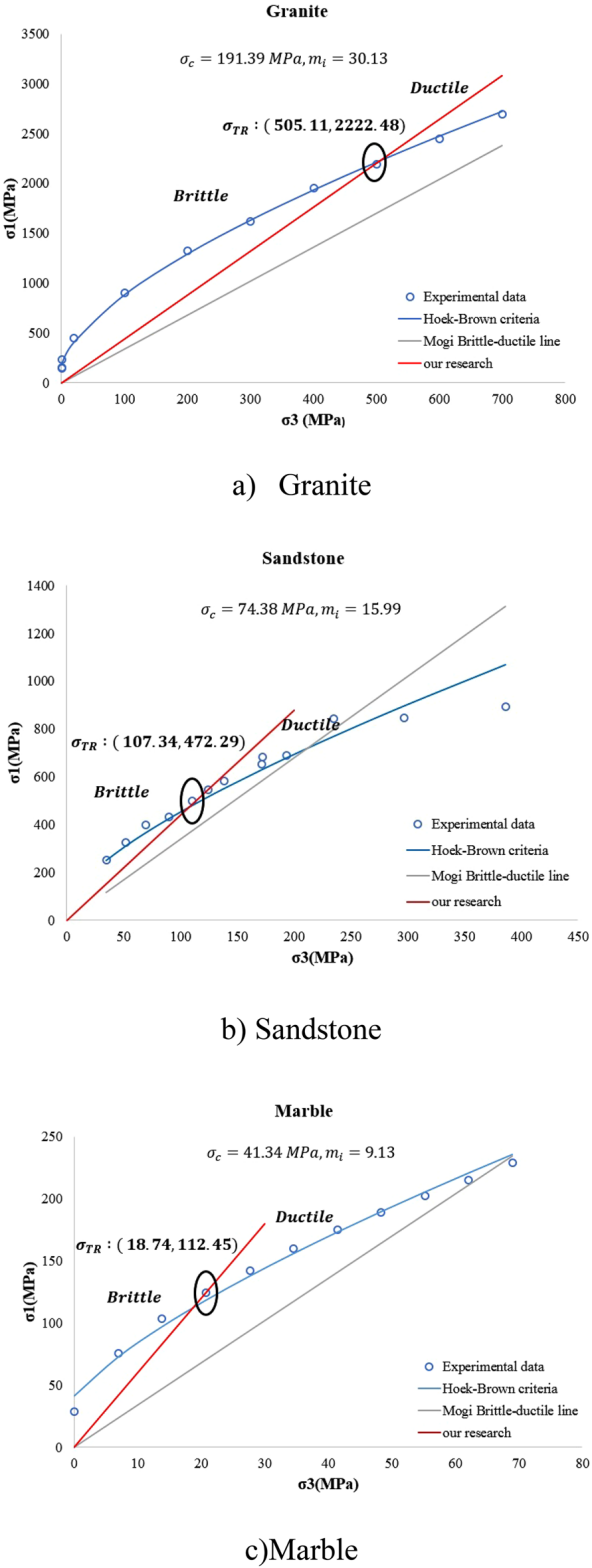
Brittle-ductile transition stress based on Eqs. (7) and (8) for (**a)** Granite, (**b**) Sandstone, and (**c**) Marble.

Based on Eqs. (7) and (8), the value of σ_TR_ is influenced non-linearly by the value of mi. In other words, as mi increases, σ_TR_ increases. Equations (7) and (8) are in good agreement with the empirical failure criterion proposed by Mogi^2^ which suggests that by increasing the rigidity of rock, the required confining pressure ${\upsigma }_{3}$ that triggers brittle-ductile transition increases. In the same way, Tsikrikis et al.^50^ performed a set of triaxial compressive tests on low-porosity carbonated rocks and observed that the σ_TR_ decreases logarithmically with decreasing m_i_, increasing the average rock grain size and decreasing the ratio of the σ_TR_ to the unconfined compressive strength $\frac{{\sigma }_{tr}}{{\sigma }_{c}}$, but the stress ratio ($\frac{{\sigma }_{1}}{{\sigma }_{3}}$) is approximately the same and independent of rock type, grain size, σ_TR,_ and m_i_. Based on their analysis, it was found that σ_TR_ can be formulated as a function of mi and ${\sigma }_{c}$ with the coefficient of determination of *R*^2^ = 0.9, which shows good agreement with our research findings with respect to the relationship between m_i_ and the ratio between transition stress and uniaxial compressive strength ($\frac{{\sigma }_{tr}}{{\sigma }_{c}}$). Tsikrikis et al.^50^, conducted experimental tests over limestone and calculated m_i_ = 23.5 and UCS = 66.6 MPa. Based on their measurements, the value of σ_TR_ was 63 MPa. While using the proposed equation in this research [Eq. (8)] to calculate the σ_TR,_ its value is 64 MPa which means that the results are close to each other (the data are summarized in Table A.4). On the other hand, for marble, they found the value of σ_TR_ was 23.8 MPa; however, according to our formula, the value of σ_TR_ is 28.3 MPa, which shows some discrepancies between the prediction of our model and their observation. Figure 9 shows the brittle, ductile, and brittle-ductile regions based on our proposed model [Eqs. (7) and (8)]. Compared with Hoek–Brown failure criteria, Mogi brittle-ductile transition stress, and experimental data for granite, sandstone, and marble (see Table A.4). For granite, with UCS = 191.39 MPa and m_i_ = 30.13, the brittle region is between 0 and ${\sigma }_{3}$=505 MPa, the ductile region occurs at ${\sigma }_{3}$ > 505.11, and the brittle-ductile region occurs at σ_TR_ = 505.11 MPa. For sandstone, with UCS = 74.38 MPa and m_i_ = 15.99, the brittle region is between 0 and ${\sigma }_{3}$=107.34 MPa, the ductile region occurs at ${\sigma }_{3}$ > 107.34, and the brittle-ductile region occurs at σ_TR_ = 107.34 MPa. For marble, with UCS = 41.34 MPa and m_i_ = 9.13, the brittle region is between 0 and ${\sigma }_{3}$=41.34 MPa, the ductile region occurs at ${\sigma }_{3}$ > 41.34, and the brittle-ductile region occurs at σ_TR_ = 41.34 MPa. The rocscience program^51^ was used for the calculations.

Similarly, Zuo and Shen^48^ proposed a micromechanics-based frictional damage model to investigate the brittle-ductile transition process of various rocks and found that critical damage at failure can be linearly related to the level of confining pressure. The amount of ductile deformation and the strength increase progressively with increasing confining pressure until fully ductile deformation occurs with apparent work-hardening. This result can be linked to the micro-mechanics principle of m_i_ conducted by Hoek and Martin^52^, which incorporates the role of coefficient of friction for pre-existing sliding crack surfaces and an intermediate fracture mechanics parameter that can be obtained from experimental data. They conclude that as the ratio of the coefficient of friction to the intermediate fracture parameter increase, the value of m_i_ increases.

Walton^29^ analyzed the large database for different rock types. Based on his analysis, transition stress (σ_TR_) depends on ductility parameter (*d*), UCS, and Hoek–Brown material constant (*m*_*i*_). Through re-interpretation of previously published stress–strain data for a wide variety of rocks, silicate rocks (*d*) vary from 2.5 to 3, and for carbonate rocks is between 3.5 and 5, which is in good agreement with our results. Similarly, Iyare et al.^47^ developed the experimental model based on a set of triaxial tests on mudstone samples to predict the σ_TR_ They observed that for the tested samples, σ_TR_ varies between 50 and 90 MPa, which is in good agreement with our proposed formula (Fig. 7) for the determination of σ_TR_ based on UCS for sedimentary rocks with the coefficient of determination (*R*^2^ = 0.7).

It is worth mentioning that more detailed material models beyond ideal elasticity give an exact relationship between rock strength parameters such as uniaxial compressive strength, *m*_*i,*_ and σ_TR_ Notably, the observed relations can be explained in a universal thermodynamic framework where internal variables characterise the structural changes^53,54^. These constitutive models are based only on universal principles of thermodynamics, are independent of particular mechanisms, and are successful in characterizing rheological phenomena in the brittle-ductile transition region of rocks, including and beyond simple creep and relaxation. This is in accordance with the difficulty of finding a very detailed quantitative mesoscopic mechanism for the brittle-ductile phenomena in rocks^55,56^.

## Conclusions

This study represents an investigation of a large database of compression tests performed on different kinds of rocks over a wide range of confining stresses. The regression analyses of the relationships between uniaxial compressive strength (UCS), Hoek–Brown material constant (*m*_*i*_), and brittle-ductile transition stress (σ_TR_) showed that there is a new nonlinear correlation between uniaxial compressive strength and transition stress. This research reveals that the relation between the σ_TR_ and UCS and *m*_i_ is rock-type dependent. It means that for different rock types, the proposed formula has different material coefficients. For silicate rock (Granite), the slope of the brittle-ductile transition stress line, which we obtained in this research, is less than the slope of the brittle-ductile transition stress line for carbonate rocks (Marble). In other words, for silicate rock, the slope of the brittle-ductile transition line is shown by ${\sigma }_{1}$= 4.4 ${\sigma }_{3}$; whereas for carbonated rocks in this research, the slope of the brittle-ductile transition line is shown by ${\sigma }_{1}$= 6 ${\sigma }_{3}$ Regression analyses show that the determination coefficient between σ_TR_ and UCS for gneiss is 0.9, sandstone is 0.8, and shale is 0.74. Similarly, the determination coefficient between ${\sigma }_{TR}$ and *m*_i_ for gneiss is 0.88. Based on the regression analysis and due to the high determination coefficient between UCS and σ_TR_ for different rocks, the UCS can be considered a significant parameter to estimate the σ_TR_. In addition, for the igneous rocks, both *m*_i_ and UCS can be used for suggesting the σ_TR_. The result of this research can be used to estimate σ_TR_ for different rock types in engineering practice. Future work should expand on the analyses presented in this paper, mainly focusing more on metamorphic rocks and considering the influences of fluid saturation and proper triaxial loading conditions on the brittle-ductile transition.

## Supplementary Information

Supplementary Tables.

## Data Availability

The datasets generated and/or analyzed during the current study are available in the book of Sheorey^49^.

## References

[1] Heard HC, Griggs D, Handin J (1960). Transition from brittle fracture to ductile flow in Solnhofen limestone as a function of temperature, confining pressure, and interstitial fluid pressure. Rock deformation.

[2] Mogi K (1966). Pressure dependence of rock strength and transition from brittle fracture to ductile flow. Bull. Earthq. Res. Inst..

[3] Mogi K (1972). Fracture and flow of rocks. Dev. Geotect..

[4] Byerlee JD (1968). Brittle-ductile transition in rocks. J. Geophys. Res..

[5] Evans B, Fredrich J, Wong TF (1990). The brittle-ductile transition in rocks: Recent experimental and theoretical progress. Geophys. Monogr. Ser..

[6] Jaeger JC, Cook NGW, Zimmerman RW (2007). Fundamentals of Rock Mechanics.

[7] Wong T-F, Baud P (2012). The brittle-ductile transition in porous rock: A review. J. Struct. Geol..

[8] Schopfer MPJ, Childs C, Manzocchi T (2013). Three-dimensional failure envelopes and the brittle-ductile transition. J. Geophys. Res. Solid Earth.

[9] Lyakhovsky V, Zhu W, Shalev E (2015). Visco-poroelastic damage model for brittle-ductile failure of porous rocks. J. Geophys. Res. Solid Earth.

[10] Liu W, Zhu X, Jing J (2018). The analysis of ductile-brittle failure mode transition in rock cutting. J. Petr. Sci. Eng..

[11] Aharonov E, Scholz CH (2019). The brittle-ductile transition predicted by a physics-based friction law. J. Geophys. Res. Solid Earth.

[12] Zhao J, Feng X-T, Zhang X, Yang C (2019). Brittle and ductile creep behavior of Jinping marble under true triaxial stress. Eng. Geol..

[13] Liu SL, Chen HR, Yuan SS, Zhu QZ (2020). Experimental investigation and micromechanical modeling of the brittle-ductile transition behaviors in low-porosity. Int. J. Mech. Sci..

[14] Davarpanah M, Somodi G, Vásárhelyi B (2020). Experimental determination of the mechanical properties and deformation constants of Mórágy granitic rock formation (Hungary). Geotech. Geol. Eng..

[15] You T, Waisman H, Zhu QZ (2021). Brittle-ductile failure transition in geomaterials modeled by a modified phase-field method with a varying damage-driving energy coefficient. Int. J. Plast..

[16] Jacquey AB, Cacace M (2020). Multiphysics modeling of a brittle-ductile lithosphere: 2. Semi-brittle, semi-ductile deformation and damage rheology. J. Geophys. Res. Solid Earth.

[17] Su C, Qiu J, Wu Q, Weng L (2020). Effects of high temperature on the microstructure and mechanical behavior of hard coal. Int. J. Min. Sci. Technol..

[18] Kim B-H, Larson MK (2021). Laboratory investigation of the anisotropic confinement-dependent brittle-ductile transition of a Utah coal. Int. J. Min. Sci. Technol..

[19] John ML (2008). Porosity and the brittle-ductile transition in sedimentary rocks. AIP Conf. Proc.

[20] Kármán T (1910). Mitől függ az anyag igénybevétele? (What influences the strength of the materials?). Magyar Mérnök Egylet Közlönye.

[21] Kármán T (1911). Festigkeits Versuche unter allseitigem Druck. Z. Verhandl. Deut. Ingr..

[22] Ledniczky K, Vásárhelyi B (2000). Brittle-ductile transition of anisotropic rocks during three-point bending test. Acta Geod. Geophys. Hung..

[23] Vásárhelyi B (2010). Tribute to the first triaxial test performed in 1910. Acta Geod. Geophys. Hung..

[24] Ván P, Vásárhelyi B, Zhao J, Labiouse V, Dudt JP, Mathier JF (2010). Centenary of the first triaxial test - recalculation of the results of Kármán. Eurock'2010 (Laussane), Rock Mech, in Civil and Environment.

[25] Deák F, Ván P, Vásárhelyi B (2012). Hundred years after the first triaxial test. Period. Polytech. Civil. Eng..

[26] Erarslan, N. & Ghamgosar, M. Fracturing and indirect tensile strength of brittle and ductile rocks. *2014 ISRM European Regional Symposium on Rock Engineering and Rock Mechanics: Structures in and on Rock Masses, EUROCK 2014*, 321–324 (2014).

[27] Paterson MSM, Wong T-F (2005). Experimental Rock Deformation: The Brittle Field.

[28] Wang S, Yang SQ (2022). A new constitutive model capturing brittle–ductile transition for crystalline marble. Arab. J. Geosci..

[29] Walton G (2021). A new perspective on the brittle-ductile transition of rocks. Rock Mech. Rock Eng..

[30] Feng XT, Zhang CQ, Qiu SL, Zhou H, Jiang Q, Li SJ (2016). Dynamic design method for deep hard rock tunnels and its application. J. Rock Mech. Geotech. Eng..

[31] Walton G, Arzúa J, Alejano LR, Diedrichs MS (2015). A laboratory-testing-based study on the strength, deformability, and dilatancy of carbonate rocks at low confinement. Rock Mech. Rock. Eng..

[32] Liu Z, Shao J (2017). Strength behavior, creep failure and permeability change of a tight marble under baud triaxial compression. Rock Mech. Rock Eng..

[33] Schlumberger. Technical challenges-carbonate reservoirs. https://www.slb.com/technical-challenges/carbonates. Accessed 11 April 2021

[34] Hoek E, Brown ET (1997). Practical estimates of rock mass strength. Int. J. Rock Mech. Min. Sci..

[35] Hoek E, Brown ET (2019). The Hoek–Brown failure criterion and GSI: 2018 edition. J. Rock Mech. Geotech. Eng..

[36] Mogi K (2007). Experimental Rock Mechanics: 3 Geomechanics Research Series.

[37] Baud P, Hall S, Heap MJ, Ji Y, Wong T-F (2021). The brittle-ductile transition in porous limestone: Failure mode, constitutive modeling of inelastic deformation and strain localization. J. Geophys. Res. Solid Earth.

[38] Wang S, Zhao W, Fu X, Zhang Z, Wang T, Ge J (2020). A universal method for quantitatively evaluating rock brittle-ductile transition behaviors. J. Petr. Sci. Eng..

[39] Davarpanah SM, Sharghi M, Vásárhelyi B, Török Á (2021). Characterization of Hoek–Brown constant m_i_ of quasi-isotropic intact rock using rigidity index approach. Acta Geotech..

[40] Hoek E, Brown ET (1980). Underground Excavation in Rock.

[41] Eberhardt E (2012). The Hoek–Brown failure criterion. Rock Mech. Rock Eng..

[42] Singh M, Raj A, Singh B (2011). Modified Mohr-Coulomb criterion for nonlinear triaxial and polyaxial strength of intact rocks. Int. J. Rock Mech. Mining Sci..

[43] Peng J, Rong G, Cai M, Wang X, Zhou C (2014). An empirical failure criterion for intact rocks. Rock Mech. Rock Engng..

[44] Schwartz, A.E. Failure of rock in the triaxial shear test. In: *Proc. 6th Rock Mech. Symp. Rolla*, 109–151 (University of Missouri, 1964)

[45] Herrmann J, Rybacki E, Sone H, Dresen G (2018). Deformation experiments on bowland and posidonia shale—part i: Strength and young's modulus at ambient and in situ pc–T conditions. Rock Mech. Rock Eng..

[46] Nicolas A, Fortin J, Regnet JB, Verberne BA, Plümper O, Dimanov A, Spiers CJ, Guéguen Y (2017). Brittle and semibrittle creep of tavel limestone deformed at room temperature. J. Geophys. Res. Solid Earth.

[47] Iyare UC, Blake OO, Ramsook R (2021). Modelling the failure behaviour of mudstones under high pressures. Rock Mech. Rock Eng..

[48] Zuo J, Shen J (2020). The Hoek–Brown Failure Criterion—From Theory to Application.

[49] Sheorey PR (1997). Empirical Rock Failure Criteria.

[50] Tsikrikis A, Papaliangas T, Marinos V (2022). Brittle-ductile transition and Hoek–Brown mi constant of low-porosity carbonate rocks. Geotech. Geol. Eng..

[51] Rocscience RocData. Version 5.0, Rocscience Inc. www.rocscience.com (2015)

[52] Hoek E, Martin CD (2014). Fracture initiation and propagation in intact rock: a review. J. Rock Mech. Geotech. Eng..

[53] Asszonyi C, Fülóp T, Ván P (2015). Distinguished rheological models for solids in the framework of a thermodynamical internal variable theory. Contin. Mech. Thermodyn..

[54] Berezovski A, Ván P (2017). Internal Variables in Thermoelasticity.

[55] Barnaföldi GG (2017). First report of long term measurements of the MGGL laboratory in the Mátra mountain range. Class. Quant. Grav..

[56] Ván P, Barnaföldi GG, Bulik T (2019). Long term measurements from the Mátra gravitational and geophysical laboratory. Eur. Phys. J. Spec. Top..

