# Supplementary material for: Brittle-ductile transition stress of different rock types and its relationship with uniaxial compressive strength and Hoek–Brown material constant (mi)
Source: Sci Rep. 2023 Jan 21;13:1186. doi: 10.1038/s41598-023-28513-3 (PMC9867716; doi:10.1038/s41598-023-28513-3)
Supplement: Supplementary file 1 — Supplementary Tables. [file 41598_2023_28513_MOESM1_ESM.docx]

**Appendix A**

**Table A.1.** Published Values of Triaxial Parameters for Hoek-Brown Criterion Using Data Set for Igneous Rocks with the Calculated Transition Stress (Sheorey, 1997).

| **No.** | **Rock name** | $\sigma_{c}$ **(MPa)** | $\sigma_{t}$ **(MPa)** | ***m_i_*** | $\sigma_{TR}$ |
| --- | --- | --- | --- | --- | --- |
| 1 | agglomerate tuff | 92 | 11.43 | 7.926 | 73.1 |
| 2 | andesite | 201.9 | 31.64 | 6.225 | 134.87 |
| 3 | basalt | 79.1 | 17.45 | 4.313 | 42.31 |
| 4 | diabase | 322.9 | 15.85 | 20.324 | 583.17 |
| 5 | diabase | 532.0 | 34.60 | 15.310 | 737.76 |
| 6 | diorite | 67.8 | 10.82 | 6.103 | 44.69 |
| 7 | diorite | 124.3 | 18.57 | 6.548 | 85.96 |
| 8 | gabbro | 379.1 | 25.10 | 15.033 | 517.04 |
| 9 | gabbro | 226.9 | 10.92 | 20.738 | 417.71 |
| 10 | granite | 241.3 | 11.34 | 21.227 | 454.18 |
| 11 | granite | 318.2 | 10.31 | 30.816 | 858.44 |
| 12 | granite | 260.0 | 18.56 | 13.936 | 331.1 |
| 13 | granite breccia | 334.9 | 21.06 | 15.837 | 479.06 |
| 14 | granodiorite | 113.1 | 10.16 | 11.043 | 117.46 |
| 15 | granodiorite | 259.1 | 14.23 | 18.15 | 420.61 |
| 16 | lamprophyre | 116.3 | 14.02 | 8.174 | 94.6 |
| 17 | quartzdiorite | 174.7 | 9.53 | 18.274 | 285.42 |
| 18 | quartzdiorite | 173.4 | 14.47 | 11.903 | 192.09 |
| 19 | quartzdiorite | 98.6 | 7.35 | 13.343 | 120.77 |
| 20 | quartzdiorite | 273.8 | 15.71 | 17.371 | 426.63 |
| 21 | quartzdiorite | 209.7 | 9.98 | 20.965 | 390.06 |
| 22 | rhyolite | 106.4 | 18.96 | 5.430 | 65.04 |

**Table A.2.** Published Values of Triaxial Parameters for Hoek-Brown Criterion Using Data Set for Sedimentary Rocks with the Calculated Transition Stress Stress (Sheorey, 1997).

| **No.** | **Rock name** | $\sigma_{c}$ **(MPa)** | $\sigma_{t}$ **(MPa)** | ***m_i_*** | $\sigma_{TR}$ |
| --- | --- | --- | --- | --- | --- |
| 1 | dolomite | 145.3 | 18.2 | 7.859 | 114.7 |
| 2 | dolomite | 524.5 | 64.22 | 8.044 | 421.44 |
| 3 | limestone | 65.9 | 4.47 | 14.663 | 87.86 |
| 4 | limestone | 128.8 | 9.85 | 12.992 | 154.07 |
| 5 | limestone | 94.9 | 13.15 | 7.076 | 69.33 |
| 6 | limestone | 53.6 | 7.84 | 6.686 | 37.61 |
| 7 | sandstone | 85.2 | 9.87 | 8.52 | 71.57 |
| 8 | sandstone | 75.5 | 8.72 | 8.543 | 63.55 |
| 9 | sandstone | 149.9 | 6.51 | 22.996 | 304.57 |
| 10 | sandstone | 129.9 | 18.15 | 7.017 | 94.33 |
| 11 | sandstone | 112.9 | 14.68 | 7.561 | 86.58 |
| 12 | sandstone | 109.0 | 8.11 | 13.367 | 133.72 |
| 13 | sandstone | 21.7 | 0.88 | 24.537 | 46.93 |
| 14 | sandstone | 152.4 | 16.54 | 9.11 | 134.98 |
| 15 | sandstone | 74.2 | 5.98 | 12.33 | 84.76 |
| 16 | sandstone | 300.2 | 23.57 | 12.658 | 350.93 |
| 17 | sandstone | 74.6 | 4.28 | 17.378 | 116.29 |
| 18 | sandstone | 94.3 | 12.13 | 7.652 | 72.96 |
| 19 | sandstone | 211.7 | 18.09 | 11.618 | 229.64 |
| 20 | sandstone | 41.5 | 2.92 | 14.123 | 53.49 |
| 21 | sandstone | 217.9 | 39.62 | 5.319 | 131.5 |
| 22 | sandstone | 91.2 | 10.56 | 8.525 | 76.64 |
| 23 | sandstone | 65.4 | 5.79 | 11.206 | 68.78 |
| 24 | sandstone | 93.9 | 3.78 | 24.761 | 204.85 |
| 25 | sandstone | 42.6 | 1.22 | 35.014 | 130.24 |
| 26 | sandstone | 150.6 | 14.8 | 10.079 | 144.85 |
| 27 | sandstone | 75.4 | 5.25 | 14.288 | 98.2 |
| 28 | sandstone | 93.3 | 9.74 | 9.474 | 85.29 |
| 29 | sandstone | 10.0 | 0.4 | 25.314 | 22.29 |
| 30 | sandstone | 220.6 | 8.28 | 26.589 | 515.56 |
| 31 | sandstone | 14.1 | 0.93 | 15.1232 | 19.34 |
| 32 | sandstone | 23.6 | 2.26 | 10.334 | 23.18 |
| 33 | sandstone | 58.9 | 13.27 | 4.213 | 31.11 |
| 34 | sandstone | 36.5 | 4.13 | 8.728 | 31.25 |
| 35 | sandstone | 30.3 | 3.45 | 8.673 | 25.81 |
| 36 | sandstone | 40.1 | 3.96 | 10.034 | 38.43 |
| 37 | sandstone | 28.2 | 1.63 | 17.3 | 43.77 |
| 38 | sandstone | 26.2 | 2.1 | 12.401 | 30.08 |
| 39 | sandstone | 10.8 | 0.59 | 18.403 | 17.76 |
| 40 | sandstone | 10.6 | 0.38 | 28.021 | 26.07 |
| 41 | sandstone | 32.3 | 3.17 | 10.101 | 31.12 |
| 42 | sandstone | 31.3 | 3.47 | 8.915 | 27.25 |
| 43 | sandstone | 18.7 | 1.46 | 12.763 | 22.02 |
| 44 | sandstone | 15.6 | 0.61 | 25.643 | 35.2 |
| 45 | sandstone | 35.6 | 3.97 | 8.871 | 30.87 |
| 46 | sandstone | 33.0 | 3.13 | 10.42 | 32.63 |
| 47 | sandstone | 38.0 | 4.13 | 9.09 | 33.6 |
| 48 | sandstone | 17.2 | 1.33 | 12.94 | 20.5 |
| 49 | sandstone | 19.6 | 1.35 | 14.483 | 25.84 |
| 50 | sandstone | 32.5 | 3.29 | 9.784 | 30.5 |
| 51 | sandstone | 25.1 | 2.12 | 11.792 | 27.58 |
| 52 | sandstone | 28.9 | 1.87 | 15.382 | 40.25 |
| 53 | sandstone | 65.2 | 6.73 | 9.582 | 60.16 |
| 54 | sandstone | 132.0 | 21.18 | 6.075 | 86.74 |
| 55 | sandstone | 45.0 | 6.26 | 7.038 | 32.75 |
| 56 | sandstone | 54.7 | 9.3 | 5.714 | 34.53 |
| 57 | sandstone | 58.7 | 10.63 | 5.345 | 35.53 |
| 58 | sandstone | 58.0 | 9.56 | 5.905 | 37.41 |
| 59 | sandstone | 9.8 | 0.24 | 40.725 | 34.76 |
| 60 | sandstone | 63.4 | 8.65 | 7.197 | 46.89 |
| 61 | sandstone | 272.8 | 11.32 | 24.061 | 578.93 |
| 62 | sandstone | 234.8 | 7.8 | 30.063 | 618.34 |
| 63 | sandstone | 222.2 | 7.54 | 29.438 | 573.29 |
| 64 | sandstone | 224.8 | 7.66 | 29.301 | 577.37 |
| 65 | sandstone | 212.5 | 6.57 | 32.329 | 600.78 |
| 66 | sandstone | 252.8 | 9.7 | 26.014 | 578.44 |
| 67 | sandstone | 254.2 | 10.93 | 23.229 | 521.52 |
| 68 | sandstone | 226.4 | 7.21 | 31.387 | 621.84 |
| 69 | sandstone | 267.0 | 8.98 | 29.7 | 694.85 |
| 70 | sandstone | 163.0 | 10.41 | 15.602 | 229.99 |
| 71 | sandstone | 49.2 | 6.09 | 7.957 | 39.21 |
| 72 | sandstone | 173.7 | 14.9 | 11.57 | 187.75 |
| 73 | sandstone | 236.1 | 56.05 | 3.975 | 121.03 |
| 74 | sandstone | 193.1 | 13.83 | 13.884 | 245.08 |
| 75 | sandstone | 115.7 | 9.83 | 11.681 | 126.09 |
| 76 | sandstone | 76.9 | 6.26 | 12.191 | 86.98 |
| 77 | sandstone | 72.1 | 5.48 | 13.082 | 86.77 |
| 78 | sandstone | 104.6 | 16.33 | 6.251 | 70.07 |
| 79 | sandstone | 163.5 | 10.92 | 14.9 | 221.19 |
| 80 | sandstone | 110.7 | 7.26 | 15.174 | 152.27 |
| 81 | sandstone | 98.8 | 8.09 | 12.125 | 111.22 |
| 82 | sandstone | 103.6 | 10.63 | 9.643 | 96.08 |
| 83 | sandstone | 104.2 | 13.39 | 7.652 | 80.62 |
| 84 | sandstone | 44.2 | 3.39 | 12.972 | 52.8 |
| 85 | sandstone | 61.0 | 5.75 | 10.51 | 60.76 |
| 86 | sandstone | 48.2 | 2.87 | 16.759 | 72.64 |
| 87 | sandstone | 99.5 | 7.39 | 13.379 | 122.17 |
| 88 | sandstone | 162.1 | 16.47 | 9.741 | 151.59 |
| 89 | sandstone | 102.1 | 5.82 | 17.498 | 160.18 |
| 90 | sandstone | 110.3 | 6.33 | 17.382 | 171.97 |
| 91 | sandstone | 86.7 | 4.38 | 19.734 | 152.28 |
| 92 | sandstone | 279.7 | 25.21 | 11.004 | 289.61 |
| 93 | sandstone | 306.5 | 23.24 | 13.115 | 369.71 |
| 94 | sandstone | 218.7 | 12.46 | 17.493 | 343.01 |
| 95 | sandstone | 337.7 | 15.45 | 21.812 | 652.31 |
| 96 | sandstone | 72.7 | 3.8 | 19.065 | 123.6 |
| 97 | sandstone | 109.2 | 15 | 7.145 | 80.33 |
| 98 | sandstone | 28.9 | 3.93 | 7.216 | 21.41 |
| 99 | sandstone | 111.9 | 14.21 | 7.748 | 87.39 |
| 100 | sandstone | 116.4 | 5.86 | 19.813 | 205.21 |
| 101 | sandstone | 104.9 | 5.88 | 17.793 | 167.16 |
| 102 | sandstone | 119.2 | 7.18 | 16.551 | 177.59 |
| 103 | sandstone | 49.9 | 6.91 | 7.087 | 36.49 |
| 104 | sandstone | 54.8 | 1.56 | 35.107 | 167.97 |
| 105 | sandstone | 93.8 | 2.75 | 34.034 | 278.89 |
| 106 | sandstone | 17.1 | 0.97 | 17.579 | 26.94 |
| 107 | sandstone | 70.8 | 7.68 | 9.115 | 62.74 |
| 108 | sandstone | 64.8 | 5.02 | 12.824 | 76.63 |
| 109 | sandstone | 111.8 | 8.6 | 12.925 | 133.12 |
| 110 | sandstone | 62.9 | 9.64 | 6.37 | 42.68 |
| 111 | sandstone | 125.2 | 5.44 | 22.963 | 254.04 |
| 112 | sandstone | 45.0 | 3.04 | 14.754 | 60.34 |
| 113 | shale | 242.0 | 39.35 | 5.988 | 157.52 |
| 114 | shale | 181.9 | 40.14 | 4.311 | 97.26 |
| 115 | shale | 99.2 | 30.5 | 2.945 | 44.43 |
| 116 | shale | 106.3 | 31.44 | 3.085 | 48.52 |
| 117 | shale | 124.0 | 27.16 | 4.345 | 66.58 |
| 118 | shale | 162.6 | 34.66 | 4.479 | 88.77 |
| 119 | shale | 220.3 | 44.09 | 4.797 | 125 |
| 120 | shale | 220.6 | 33.85 | 6.364 | 149.59 |
| 121 | shale | 184.7 | 25.29 | 7.164 | 136.14 |
| 122 | shale | 154.0 | 17.56 | 8.655 | 130.97 |
| 123 | shale | 84.5 | 5.35 | 15.737 | 120.17 |
| 124 | shale | 185.0 | 26.47 | 6.848 | 132.02 |
| 125 | shale | 190.2 | 27.28 | 6.83 | 135.48 |
| 126 | shale | 175.0 | 21.34 | 8.078 | 141.07 |
| 127 | shale | 193.9 | 26.86 | 7.082 | 141.74 |
| 128 | shale | 112.0 | 23.52 | 4.555 | 61.71 |
| 129 | shale | 107.9 | 26.9 | 3.762 | 53.83 |
| 130 | shale | 78.8 | 15.73 | 4.808 | 44.77 |
| 131 | shale | 57.4 | 11.15 | 4.959 | 33.21 |
| 132 | shale | 66.8 | 13.15 | 4.886 | 38.31 |
| 133 | shale | 93.2 | 22.07 | 3.986 | 47.84 |
| 134 | shale | 99.3 | 21.88 | 4.318 | 53.14 |
| 135 | shale | 58.0 | 8.08 | 7.043 | 42.23 |
| 136 | shale | 80.4 | 13.76 | 5.672 | 50.52 |
| 137 | shale | 66.9 | 4.21 | 15.826 | 95.64 |
| 138 | shale | 25.9 | 2.23 | 11.509 | 27.87 |
| 139 | shale | 28.7 | 3.72 | 7.568 | 22.02 |
| 140 | shale | 100.3 | 9.45 | 10.517 | 99.96 |

**Table A.3.** Published Values of Triaxial Parameters for Hoek-Brown Criterion Using Data Set for Metamorphic Rocks with the Calculated Transition Stress Stress (Sheorey, 1997).

| **No.** | **Rock name** | $\sigma_{c}$ **(MPa)** | $\sigma_{t}$ **(MPa)** | ***m_i_*** | $\sigma_{TR}$ |
| --- | --- | --- | --- | --- | --- |
| 1 | shist | 133.6 | 6.59 | 20.246 | 240.41 |
| 2 | slate | 148.6 | 18.64 | 7.844 | 117.14 |
| 3 | slate | 108.7 | 28.66 | 3.528 | 52.60 |
| 4 | slate | 53.4 | 27.5 | 1.428 | 19.35 |
| 5 | slate | 62.3 | 21.7 | 1.98 | 24.42 |
| 6 | slate | 98.0 | 41.56 | 1.933 | 38.16 |
| 7 | slate | 129.4 | 39.96 | 2.930 | 57.84 |
| 8 | slate | 178.3 | 19.97 | 8.819 | 153.89 |
| 9 | slate | 57.8 | 1.86 | 30.965 | 156.67 |
| 10 | slate | 14.5 | 0.64 | 22.700 | 29.10 |
| 11 | slate | 44.2 | 4.6 | 9.504 | 40.51 |
| 12 | slate | 68.1 | 4.03 | 16.853 | 103.17 |
| 13 | slate | 155.1 | 10.85 | 14.217 | 201.10 |
| 14 | slate | 167.6 | 12.22 | 13.644 | 209.42 |
| 15 | gneiss | 315.1 | 17.58 | 17.865 | 504.00 |
| 16 | gneiss | 75.3 | 13.63 | 5.343 | 45.57 |
| 17 | gneiss | 221.7 | 13.61 | 16.233 | 324.43 |
| 18 | gneiss | 195.4 | 29.86 | 6.389 | 132.85 |
| 19 | gneiss | 197.7 | 22.29 | 8.759 | 169.72 |
| 20 | gneiss | 106.4 | 11.17 | 9.423 | 96.84 |
| 21 | quartzite | 144.5 | 19.28 | 7.363 | 108.66 |
| 22 | quartzite | 657.6 | 21.82 | 30.102 | 1733.95 |
| 23 | quartzite | 219.0 | 13.88 | 15.71 | 311.05 |

**Table A.4.** Stress condition for calculation of Hoek-Brown Criterion

| **No.** | **Rock name** | $\sigma_{3}$ **(MPa)** | $\sigma_{1}$ **(MPa)** | ***m_i_*** | $\sigma_{c}$(MPa) |
| --- | --- | --- | --- | --- | --- |
| 1 | Granite | 0 | 150 | 30.13 | 191.39 |
|  |  | 0.1 | 160 |  |  |
|  |  | 0.5 | 238.5 |  |  |
|  |  | 20 | 456 |  |  |
|  |  | 100 | 908 |  |  |
|  |  | 200 | 1326 |  |  |
|  |  | 300 | 1620 |  |  |
|  |  | 400 | 1954 |  |  |
|  |  | 500 | 2198 |  |  |
|  |  | 600 | 2450 |  |  |
|  |  | 700 | 2700 |  |  |
| 2 | Sandstone | 34.5 | 255 | 15.99 | 74.38 |
|  |  | 51.7 | 326 |  |  |
|  |  | 68.9 | 401 |  |  |
|  |  | 89.6 | 433 |  |  |
|  |  | 110.3 | 502 |  |  |
|  |  | 124 | 548.8 |  |  |
|  |  | 138 | 586 |  |  |
|  |  | 171 | 655 |  |  |
|  |  | 172 | 686 |  |  |
|  |  | 193 | 691 |  |  |
|  |  | 234.5 | 844 |  |  |
|  |  | 296.5 | 848 |  |  |
|  |  | 386.2 | 896 |  |  |
| 3 | Marble | 0 | 28.9 | 9.13 | 41.34 |
|  |  | 6.89 | 75.8 |  |  |
|  |  | 13.79 | 104.1 |  |  |
|  |  | 20.68 | 124.8 |  |  |
|  |  | 27.58 | 142.7 |  |  |
|  |  | 34.48 | 160 |  |  |
|  |  | 41.37 | 175.2 |  |  |
|  |  | 48.27 | 189.6 |  |  |
|  |  | 55.17 | 202.7 |  |  |
|  |  | 62.07 | 215.2 |  |  |
|  |  | 68.96 | 229.6 |  |  |
